# The Development and Application of an Intelligent Assessment and Strategy Implementation System for Non-Intellectual Factors in Mathematics Learning Among Senior High School Students

**DOI:** 10.3390/jintelligence12120126

**Published:** 2024-12-11

**Authors:** Yueyuan Kang, Guangming Wang, Luxuan Liu, Jing Liu, Qianqian Gao

**Affiliations:** 1 Faculty of Education, Tianjin Normal University, Tianjin 300387, China; liujing_9508@163.com (J.L.); 2 Tianjin Academy of Educational Science, Tianjin 300191, China

**Keywords:** non-intellectual factors in mathematics, Intelligent Assessment and Strategy Implementation System, high school students, norms, mathematical achievement, evidence-based mathematics education research

## Abstract

Non-intellectual factors in mathematics are key psychological factors that influence students’ cognitive activities and, consequently, their learning efficiency. While the assessment of these factors has gained increasing academic attention, research on the effective use of intelligent tools to assess and improve students’ non-intellectual factors remains insufficient. This study employed intelligent technology to develop the Intelligent Assessment and Strategy Implementation System for Non-intellectual Factors in Mathematics Learning for Primary and Secondary School Students, which integrates an assessment index system, scales, regional norms, and personalized improvement strategies, enabling it to automatically generate bulk reports on students’ non-intellectual factor scores across various dimensions and provide targeted improvement strategies. In order to test its effectiveness, the intelligent system was applied across several provinces, cities, and schools in China. Eleventh-grade students from X Middle School in T City served as a representative case study. The interventions were based on the strategies provided by the system, and the research consistently demonstrated that the “Intelligent Assessment and Strategy Implementation System of Mathematics Non-intellectual Factors for Primary and Secondary School Students” effectively delivers high-precision diagnoses and personalized intervention strategies.

## 1. Introduction

The PISA 2018 results show that while Chinese students excel in math, they also spend the most hours on academic work of students from all participating countries. These results reflect the fact that Chinese primary and secondary school students experience a heavy academic workload and show low learning efficiency, indicating an urgent need for research on improving the efficiency of learning mathematics. Improving learning efficiency not only enhances academic performance (Wang et al. 2022) but also provides students with more time for leisure activities, contributing to better stress management (Köslich-Strumann et al. 2023) and fostering a more balanced lifestyle.

Non-intellectual factors comprise all the psychological factors that influence and determine the efficiency of intellectual activities in such activities (Wang et al. 2015). Non-intellectual actors in mathematics are psychological factors that are not directly involved in mathematical cognitive activities but influence and constrain students’ intellectual activities. Such factors have a great impact on students’ mathematics learning efficiency, which is demonstrated in students’ test results in mathematics (Liu and Zheng 2024; Christodoulou et al. 2024) and their ability to solve math problems (Eysenck and García-Calvo 1992; Xu and Qi 2022).

With the continuous development of research on non-intellectual factors in mathematics, a series of influential evaluation tools have emerged (Richardson and Suinn 1972; Suinn et al. 1988; Pintrich et al. 1993; Liu and Zheng 2024). However, these assessment tools are single-item scales administered solely through paper-and-pencil tests, for which a long time is needed to collect and analyze the data and prepare reports. This task requires considerable human and material resources, and it is essential that the researchers have the ability to analyze such data properly. In addition, the available assessment tools have only been used to investigate the current status of non-intellectual factors in mathematics among students without providing norms for the levels of such factors or proposing corresponding strategies for improvement. Moreover, although research to improve these non-intellectual factors has been conducted for many years, it often has limited application without being able to cater to the differences in students at different levels. Also, the improvement strategies proposed in such research are more theoretical than practical or are too general for implementation. With the development of science and technology and the rapid growth of artificial intelligence (AI), the integration of education with AI has gained increasing attention from scholars globally (Zhang and Aslan 2021; Schiff 2022), and AI evaluation systems are also popular research areas. However, research on intelligent systems for diagnosing non-intellectual factors and implementing improvement strategies remains insufficient. Thus, this study aimed to develop an Intelligent Assessment and Strategy Implementation System for Non-intellectual Factors in Mathematics Learning in Senior High School, which could efficiently and comprehensively diagnose the levels of non-intellectual factors in mathematics among senior high school students. It was designed to be a practical system that could provide precise strategies to improve students’ levels of non-intellectual factors.

### 1.1. Literature Review

#### 1.1.1. Non-Intellectual Factors

In the 1930s, American psychologist C. P. Alexander first proposed the concept of non-intellectual factors in his 1935 article, “Intelligence, concrete and abstract.” Wechsler continued Alexander’s research by defining the concept of “non-intellectual factors” in his 1949 article, “Cognitive, conative, and non-intellective intelligence” (Wechsler 1950), marking the formal creation of the concept. Between the 1950s and the 1980s, Neisser (1963) discussed in detail the differences between AI and the human mind in his article, “The Imitation of Man by Machine”. He argued that human beings had a unique system called emotions and that human motivations were complicated and purposive, which touches upon the non-intellectual factors in human activities. Simon (1967) emphasized the influence of motivation and emotion on human intellectual activities in his paper, “Motivational and Emotional Controls of Cognition”, and proposed the theory that motivation, emotion, and information processing are associated. Norman (1980) and Piaget (1981), among others, studied non-intellectual factors even further and proposed such non-intellectual factors as motivation, interest, emotion, belief, and will.

After the 1970s, scholars began to shift from theoretical conceptual research to empirical research, seeking a theoretical model of non-intellectual factors to explain their relationship with intellectual activities, while researchers globally began to integrate the concept with teaching practice. Lehrer and Hieronymus (1977) emphasized the motivational role of non-intellectual factors on academic achievements. According to Kriegbaum et al. (2015), non-intellectual factors, such as interest in and motivation for learning, have a profound impact on the development and improvement of students’ mathematical competence. Lee and Stankov (2018) stated that the levels of students’ non-intellectual factors directly affected their classroom performance. A survey-based study by Lu and Wu (2022) found significant differences in the levels of non-intellectual factors, such as interest, motivation, and attitude toward mathematics learning, between students with low academic achievement and those with high achievement, as students with high achievement demonstrated better non-intellectual factors than their peers with low achievements. Since then, many scholars have studied the components of non-intellectual factors and their impact on academic achievements.

In summary, many scholars define non-intellectual factors by comparing and contrasting them with intellectual factors, which helps to clarify the meaning and characteristics of non-intellectual factors. Wang et al. (2015) have synthesized the research results of psychological studies on non-intellectual factors in mathematics learning in both Chinese and international academic communities, and their results have attracted great attention due to a high citation rate. Their research focused on the components of the ICME-15 Invited Lecture, finding that although non-intellectual factors in mathematics did not directly affect individuals’ intellectual levels, they undoubtedly had an impact on individuals’ cognitive process and affective experience when learning mathematics. Such factors mainly fall along five dimensions: motivation, emotion, attitude, willpower, and personality.

#### 1.1.2. The Relationship Between Non-Intellectual Factors and Academic Achievement in Mathematics

In recent years, researchers have paid increasing attention to the impact of non-intellectual factors on students’ academic performance. These factors not only influence a student’s current academic performance but are also important predictors of his or her future academic achievement and quality of life. Studies show that responsibility (Poropat 2009) and personality traits (Almlund et al. 2011) are important non-intellectual factors that influence academic achievements.

As the influence of the PISA test expands, scholars have increasingly used PISA data to explore students’ non-intellectual factors. Cheema and Galluzzo (2013) analyzed the data of 4733 students from the U.S. portion of the PISA 2003, confirming persistent racial and socioeconomic gaps in math achievement but showing that the gender gap reduced once important predictors of math achievement, such as math self-efficacy and math anxiety, were controlled for. Wang et al. (2022) compared the mathematical metacognitive characteristics of effective and ineffective mathematics learners. Effective learners were defined as those who completed more learning tasks in the same amount of time with better learning outcomes, while ineffective learners were those who, despite investing more time and effort, produced lower-quality learning outcomes and experienced fatigue during the learning process. The study found that mathematical metacognitive monitoring and mathematical metacognitive experience in the effective learners’ group had a direct effect on their math achievement, while mathematical metacognitive knowledge had an indirect effect on math achievement. Mathematical metacognitive monitoring refers to the ability of students to observe, reflect upon, and adjust their learning behaviors in mathematics. It has five sub-dimensions: orientation and planning, organization and management, monitoring and regulation, feedback and testing, and reflection and evaluation. Mathematical metacognitive experience, on the other hand, refers to students’ ability to sense their learning process in mathematics, and it includes two sub-dimensions: cognitive experience and affective experience (Wang et al. 2016). Previous research has demonstrated that, as another key variable in addition to mathematics metacognition, non-intellectual factors in mathematics have a limited direct influence on math achievement but can indirectly influence academic achievements in mathematics, such that mathematics metacognition serves as a mediator (Kang et al. 2016).

In summary, non-intellectual factors in mathematics play an indispensable role in the process of mathematics learning and contribute to improving the mathematics learning outcomes of students. This view has been widely recognized in the academic community.

#### 1.1.3. Assessment Tools for Non-Intellectual Factors in Mathematics

After the 1980s, with the development of intelligence tests, it was found that using intellectual factors to predict human activities often generated limited results. Previous studies also identified merely moderate correlations between intelligence and students’ academic activities. Thus, some scholars began to focus on the development of tools to assess non-intellectual factors.

Currently, studies on the assessment of non-intellectual factors can be categorized primarily into comprehensive and single-item scales. Among these scales, the Noncognitive Questionnaire (NCQ, Tracey and Sedlacek 1984) and the Constructive Thinking Inventory (CTI, Epstein and Meier 1989) scales were influential comprehensive scales, while the Motivated Strategies for Learning Questionnaire (MSLQ, Poropat 2009) was an instrumental single-item scale. The non-intellectual factor measurement tools mentioned above are mainly questionnaire scales, and few studies have used AI tools to implement assessments. The questionnaire method usually requires a significant investment of labor, material resources, and time. Although the questionnaire method may not be efficient in identifying the non-intellectual factors in mathematics learning, such a method and the indicators on which a questionnaire is based form the basis for the measurement of non-intellectual factors in mathematics.

Wang et al. (2015) developed the Questionnaire on Non-intellectual Characteristics of Mathematics Learning for High School Students. The questionnaire conforms to the characteristics of Chinese secondary school students’ non-intellectual factors and constitutes a reliable and valid instrument to measure the levels of Chinese secondary school students’ non-intellectual factors in mathematics. For example, the questionnaire includes items such as, “I prefer to study topics in the mathematics textbook that spark my curiosity, even if they are difficult to understand”, and “I feel worried or anxious when I think about the upcoming math lesson”. On the basis of this questionnaire, Wang et al. (2017) established norms for the levels of non-intellectual factors in mathematics among secondary school students in Tianjin. They formulated detailed criteria for level assessment and instructions for using the questionnaire, providing a standard for the assessment of non-intellectual factors in mathematics in secondary school students. Therefore, this study used this questionnaire and the norms for further research.

Compared with middle school (grades 7 to 9, for students aged 12 to 15), mathematics learning in high school (grades 10 to 12, for students aged 15 to 18) is more extensive and significantly more challenging. At the high school level, non-intellectual factors have a significant impact on students’ achievement in mathematics (Luo et al. 2008; Lew and Hwang 2019). Therefore, it is of great practical significance to be able to quickly identify the levels of non-intellectual factors in mathematics learning among high school students and adopt precise educational strategies accordingly. However, little research has applied AI technology to high school assessment and strategy development. Work in this field is thus imperative.

### 1.2. Research Questions

The purpose of this study was to develop an Intelligent Assessment and Strategy Implementation System for Non-intellectual Factors in Mathematics Learning in Senior High School and to test the effectiveness of the system by applying it at multiple schools across various provinces and cities in China. This led to the two research questions:

1. How to develop an Intelligent Assessment and Strategy Implementation System for Non-intellectual Factors in Mathematics Learning for high school students?

2. Can the Intelligent Assessment and Strategy Implementation System for Non-intellectual Factors in Mathematics Learning for high school students efficiently diagnose the levels of non-intellectual factors in mathematics learning among Chinese high school students? How effective are the improvement suggestions proposed by the system in interventions for participants?

## 2. Methodology

To address the two research questions, this section is divided into two parts: “the development of the Intelligent Assessment and Strategy Implementation System” and “the application of the Intelligent Assessment and Strategy Implementation System”.

### 2.1. Development of the Intelligent Assessment and Strategy Implementation System

#### 2.1.1. Research Design for the Development of the Intelligent Assessment and Strategy Implementation System

**Figure 1:**
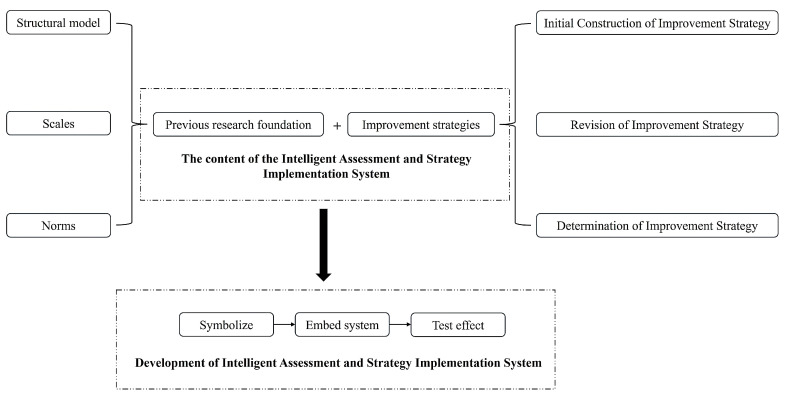
The design process of the Intelligent Assessment and Strategy Implementation System for Non-intellectual Factors in Mathematics Learning in Senior High School.

This study aims to develop an intelligent system that integrates the assessment index system, assessment scale, regional norms, and improvement strategies for the non-intellectual factors in senior high school students in mathematics. The research team previously established a comprehensive assessment index system, assessment scale (Wang et al. 2015), and regional norms (Wang et al. 2017) for the mathematics non-intellectual factors in senior high school students. Therefore, the development of the Intelligent Assessment and Strategy Implementation System is divided into two key phases: formulating improvement strategies and developing the intelligent system. The research process is outlined as follows (see Figure 1):

1. Formulation of improvement strategies: Through initial development, revision, and finalization, the improvement strategies were established to address different dimensions and proficiency levels of the non-intellectual factors in senior high school students in mathematics.

2. Development of the intelligent system: Through system integration, the assessment index system, assessment scale, regional norms, and improvement strategies were integrated into the system, enabling automatic assessment and feedback functions. Detailed procedures for development are shown below.

(1) Symbolic representation: Symbols were used to represent each dimension, the performances on each dimension, and improvement strategies.

(2) Embedding: The structural model for assessment, assessment scales, regional norms, and improvement strategies were embedded into the system.

(3) Effectiveness testing: The effectiveness of the intelligent system was tested.

#### 2.1.2. Research Tools for the Development of the Intelligent Assessment and Strategy Implementation System

In the system development process, the following tools were utilized to ensure fast and stable development outcomes and data processing:

1. Microsoft Visual Studio Community 2019: Used for developing the main interface, analysis, and reporting functions of the software.

2. NPOI plugin: Facilitates easy and efficient importation of data in Excel format and generates diagnostic reports in the same format.

3. Sunny UI: Employed for tool libraries and the processing of graphical user interfaces.

4. Database system selection: Given the limited volume of data collected at any one time, Excel was used as the database. This simplifies score calculation and results recording and minimizes back-end development work.

### 2.2. Application of the Intelligent Assessment and Strategy Implementation System

#### 2.2.1. Research Design for the Application

**Figure 2:**
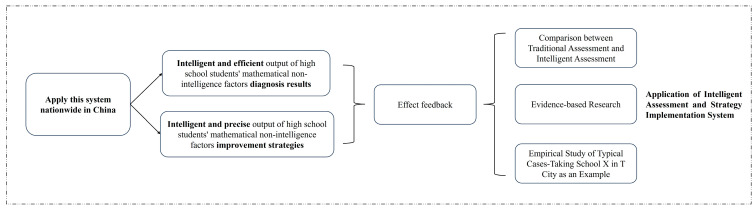
The research design for the application of the Intelligent Assessment and Strategy Implementation System for Non-intellectual Factors in Mathematics Learning in Senior High School.

First, traditional assessment methods are compared with the intelligent diagnostic effects of “The Intelligent Assessment and Strategy Implementation System of Non-intellectual Factors in Mathematics” to demonstrate the system’s efficiency in diagnosis. Then, the system is applied in multiple provinces and cities across China, and feedback is gathered to further evaluate its effectiveness. Finally, a case study is conducted in two classes at X Middle School in T City, China. These classes, which have intermediate and lower intermediate levels of non-intellectual factors in mathematics, are chosen as typical cases. Based on the intervention strategies provided by the system, improvements are implemented, thoroughly validating the effectiveness of the system. The specific research process is illustrated in Figure 2.

#### 2.2.2. Participants

The concept of Evidence-Based Education (EBE) originated in the 1990s with “evidence-based medicine” (Guyatt et al. 1992) and gradually expanded into the field of education with the promotion of the Evidence-Based Practice Movement. EBE integrates the best available scientific evidence with pedagogical expertise (Davies 1999), revealing the general laws and effectiveness of educational interventions (Slavin 2008) and providing scientific support for educational policymaking. In recent years, EBE has gained significant attention as a scientific approach in education. During the 15th International Congress on Mathematical Education (ICME-15) in July 2024, a plenary panel titled “What Counts as Evidence in Mathematics Education?” saw in-depth discussions by many scholars and experts on the importance and impact of evidence-based education in mathematics. This emphasized the critical role of evidence-based methods in improving education quality and optimizing educational decision-making.

To verify the effectiveness of the system, this study adopted a dual-method approach, combining evidence-based educational research and empirical research. This combination provides both large-scale experimental evidence and enriches micro-level empirical data through case studies in typical experimental schools, laying a scientific foundation for evaluating the system’s role in educational interventions.

1. Participants in evidence-based effectiveness testing. The Intelligent Assessment and Strategy Implementation System developed in this study has been implemented in several schools across cities and provinces in China, including T City, Q Province, B Province, Z Province, X Province, F Province, G Province, H Province, L Province, S Province, and C City. The study population consisted of senior high school students (grades 10 to 12, aged 15 to 18) from various regions and educational levels.

2. Empirical research case. This study utilized the mathematics non-intellectual factors norm for senior high school students in T City, China (Wang et al. 2017). Based on consultations with the head of the mathematics department at the experimental school, one Grade 11 (for students aged 16 or 17) class from X Middle School in T City, with an intermediate level of mathematics non-intellectual factors, was selected as Experimental Group 1. Another Grade 11 class, with lower intermediate mathematics non-intellectual factors, was selected as Experimental Group 2. A third class, with intermediate-level mathematics non-intellectual factors, served as the control group. A three-month intervention to improve the experimental groups was implemented, and no intervention was carried out for the control group. After the intervention, post-tests were conducted to assess the levels of mathematical non-intellectual factors in the intervention group, alongside the collection of materials for learning mathematics from these students. In-depth interviews were conducted with students from the experimental group who had lower pre-test scores for mathematical non-intellectual factors, as well as with their math teachers and class advisors. The empirical effects of the Intelligent Assessment and Strategy Implementation System were examined through a combination of quantitative and qualitative analyses.

#### 2.2.3. Research Tools for the Application

**Figure 3:**
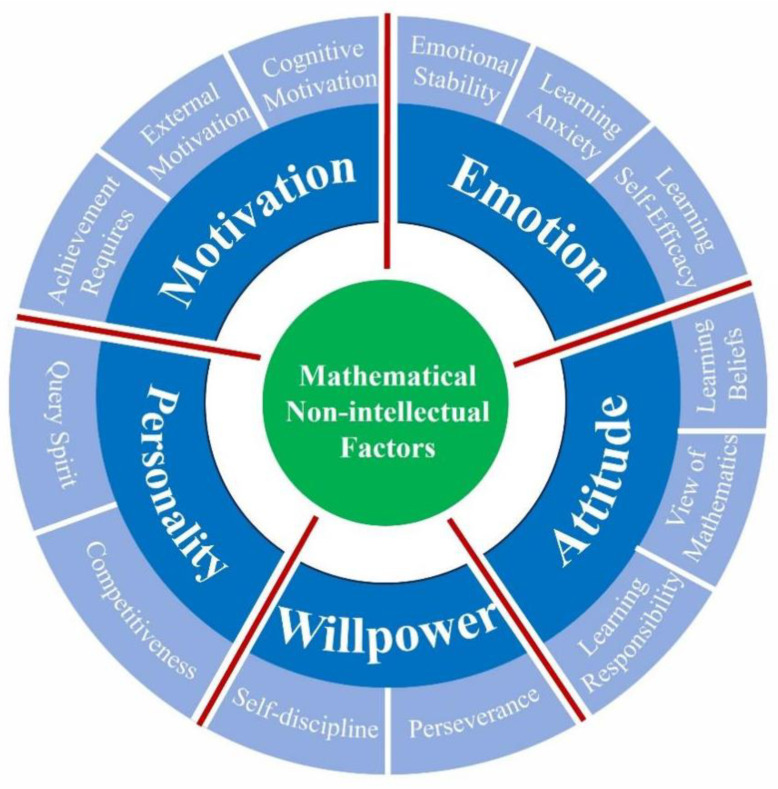
The structural model for the non-intellectual factors in mathematics learning for the high school students.

1. Questionnaire on non-intellectual factors in mathematics learning. In this study, the Questionnaire on Non-intellectual Characteristics of Mathematics Learning for High School Students (Wang et al. 2015) was selected as the assessment tool. Specifically, the structural model of non-intellectual factors in mathematics learning for high school students is shown in Figure 3. It comprises 5 dimensions (motivation, emotion, attitude, willpower, and personality) and 13 sub-dimensions (cognitive motivation, external motivation, achievement required, emotional stability, learning anxiety, learning self-efficacy, learning beliefs, view of mathematics, learning responsibility, self-discipline, perseverance, querying spirit, and competitiveness).

The analysis of the valid data presented in Table 1 shows that the Cronbach’s alpha coefficient of the overall questionnaire is 0.940, and the coefficients for individual dimensions are all above 0.7, which indicates a high degree of homogeneity for the questionnaire. The split-half reliability of the overall questionnaire is 0.955, and the split-half reliabilities for all the dimensions range from 0.764 to 0.865, indicating high internal consistency. The test-retest reliability of the overall questionnaire is 0.857, and the test-retest reliabilities of individual dimensions fall in the range of 0.788–0.812, indicating that the questionnaire has good stability. Therefore, the questionnaire has high internal and external reliabilities.

Table 2 shows a significant correlation between the dimensions of the questionnaire, with correlation coefficients ranging from 0.5 to 0.75, indicating independent relationships between the dimensions of the questionnaire. The correlation coefficients between individual dimensions and the overall questionnaire are above 0.75, which is greater than the correlation coefficients between the dimensions, indicating that the dimensions are not only relatively independent but also contribute to the whole. Thus, the overall structural validity of the questionnaire is good.

2. The “AI Batch Assessment of Mathematics Learning Quality-Non-Intelligence” of the Intelligent Assessment and Strategy Implementation System. The “AI Batch Assessment of Mathematics Learning Quality-Non-Intelligence” of the Intelligent Assessment and Strategy Implementation System used in this study is a system developed independently by our team. The system has received a patent certificate from the China National Intellectual Property Administration and a software copyright certificate (Software Copyright Registration Number: 2021SR1235186) issued by the National Copyright Administration.

The Intelligent Assessment and Strategy Implementation System for Non-intellectual Factors in Mathematics Learning is a visualized intelligent system integrating intelligent assessment, norm reference, and improvement measures, which is an efficient scientific system that provides accurate diagnoses. In the applied research of the system, two features—intelligent measurement and intelligent strategy implementation—are primarily used. In terms of intelligent assessment, the system can efficiently and comprehensively export students’ overall scores and levels as well as dimension-specific scores and performance regarding non-intellectual factors in mathematics. With respect to intelligent strategy implementation, the system can export targeted improvement strategies at corresponding levels based on the results of the assessment, providing strategic guidance and reference for the practice of educational intervention.

## 3. Results

### 3.1. Development of the Intelligent Assessment and Strategy Implementation System

**Figure 4:**
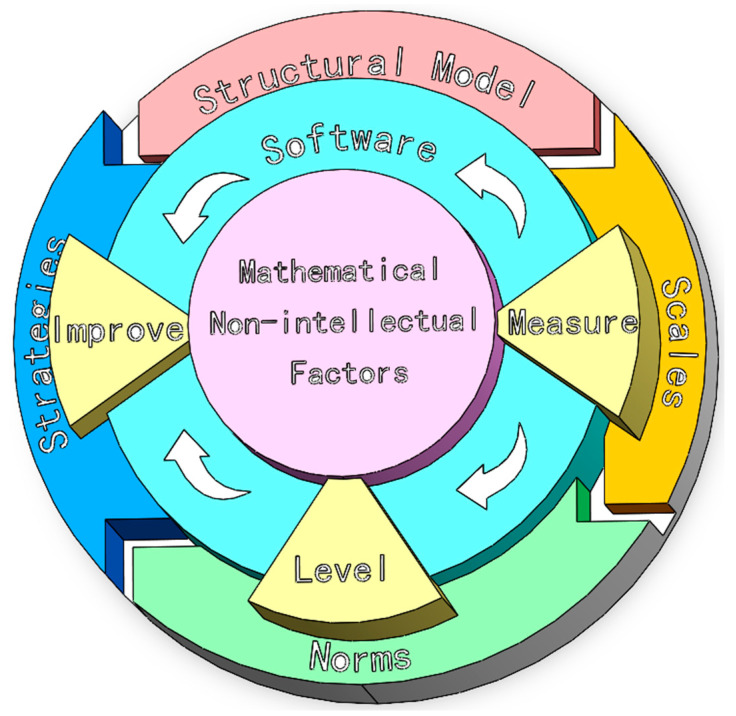
The structure of the Intelligent Assessment and Strategy Implementation System for Non-intellectual Factors in Mathematics Learning in Senior High School.

The Intelligent Assessment and Strategy Implementation System for Non-intellectual Factors in Mathematics Learning in Senior High School is an intelligent assessment software integrating an assessment index system, assessment scales, regional norms, and a personalized strategy implementation system. It is scientific and efficient and provides accurate diagnosis. The system model is shown in Figure 4. The system can measure students’ levels of non-intellectual factors in mathematics and classify these levels based on the students’ scores. It then generates personalized improvement strategies tailored to different levels. After implementing these strategies, the system is used again to reassess the students’ non-intellectual factors, forming a continuous cycle of improvement and evaluation.

The structural model, assessment scale, and regional norms are based on previous research by our team. The focus of this study lies in the development of improvement strategies and the intelligent system itself.

In terms of the development of personalized improvement strategies, this study first incorporated theoretical and empirical orientations to construct preliminary strategies to improve non-intellectual factors in mathematics learning. Theoretically, we synthesized previous studies to determine areas for improvement and major participants for strategy implementation. Empirically, we delved into the behavioral performance of outstanding students based on consultations with experts, scholars, teachers, and students with excellent levels of non-intellectual factors to ensure the practicality and relevance of the strategies. Subsequently, the improvement strategies were revised through multiple rounds by experts and teachers to enhance their logic, relevance, and precision. This process finalized the teacher and student versions of the improvement strategies for mathematics non-intellectual factors in senior high school students (see Supplementary Files S1 and S2).

#### 3.1.1. The Development Process of the Intelligent Assessment and Strategy Implementation System

This study designed and developed two versions of assessment software, one for individuals and the other for groups, covering a variety of scenarios. Both versions contain all of the features demonstrated in Figure 4 and can provide students with professional assessments and personalized advice. The difference lies in the ability of the individual version to allow students to carry out self-assessments. It provides instant feedback on assessment results and improvement strategies, as it focuses on students’ self-growth. Conversely, the group-oriented version caters to groups and exports visualized assessment results and improvement strategies for all students in a region, school, or class within a short period of time to facilitate the overall enhancement of a group of students.

The Intelligent Assessment and Strategy Implementation System of this study was developed using Microsoft Visual Studio Community 2019 tools and NPOI plug-ins, combined with SunnyUI for interface improvement. It covers the assessment functions of the three scales for elementary, middle, and high school students. To illustrate, let us examine the assessment and strategy implementation for non-intellectual factors in mathematics among high school students to understand the specific development procedures.

First, symbols were applied to represent the dimensions and their sub-dimensions. For example, A = motivation, A1 = Cognitive Motivation, etc. The specific coding is shown in Table 3 below.

Second, symbols were used to indicate the specific performance and suggestions for each dimension. For example, H-A1 refers to student performance at a high level of cognitive motivation, S-A1 refers to suggestions for students with high levels of cognitive motivation, M-A1 refers to student performance at a medium level of cognitive motivation, L-A1 means student performance at a low level of cognitive motivation, and S-A2 means suggestions made for students with medium and low levels of cognitive motivation. Please refer to Table 4 for the specific coding.

Third, the assessment index system, assessment scales, regional norms, and improvement strategies were embedded into the system according to the above coding.

Fourth, the effectiveness of the software was tested.

#### 3.1.2. Functions of the Intelligent Assessment and Strategy Implementation System

The intelligent assessment and strategy implementation software developed in this study is divided into three modules that perform basic information collection, scale data collection, and results and suggestions export. Assessors can view the overall scores for non-intellectual factors in mathematics, scores for dimensions, scores for sub-dimensions, and norm-referenced regional levels on different screens and obtain improvement strategies for the current level. The group-based batch assessment system can automatically export the assessment results and improvement strategies for non-intellectual factors in mathematics in bulk, according to the hierarchy of “school-grade-class-individual”. Specific instructions for use are provided below.

**Figure 5:**
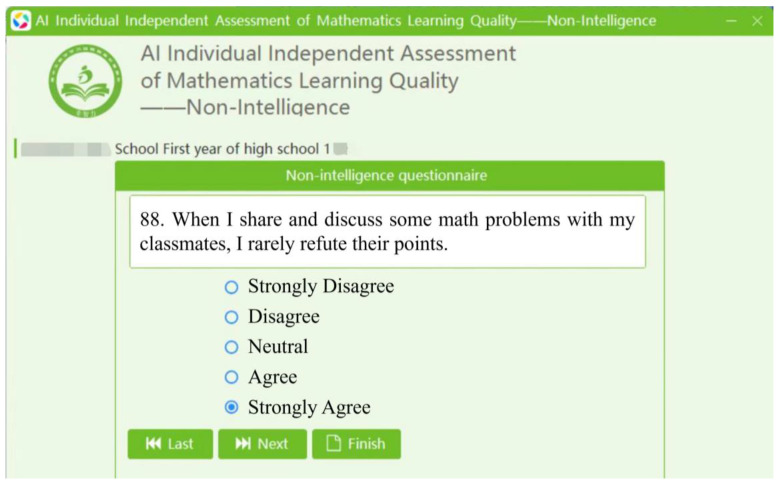
The questionnaire interface of the self-assessment and strategy implementation system.

**Figure 6:**
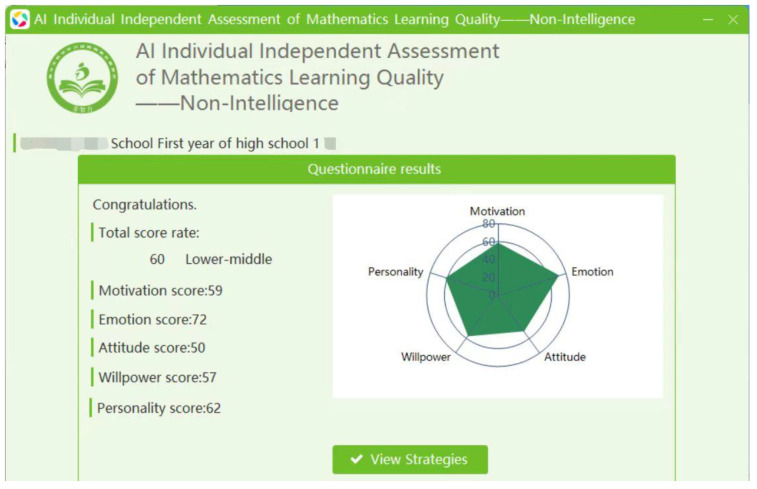
The results interface of the self-assessment and strategy implementation system.

**Figure 7:**
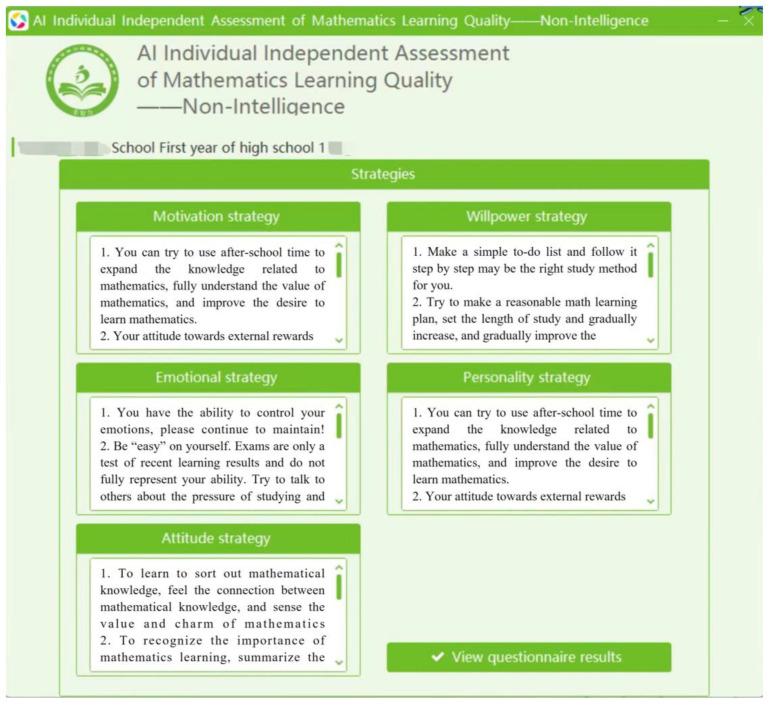
Example of the strategies interface of the self-assessment and strategy implementation system.

**Figure 8:**
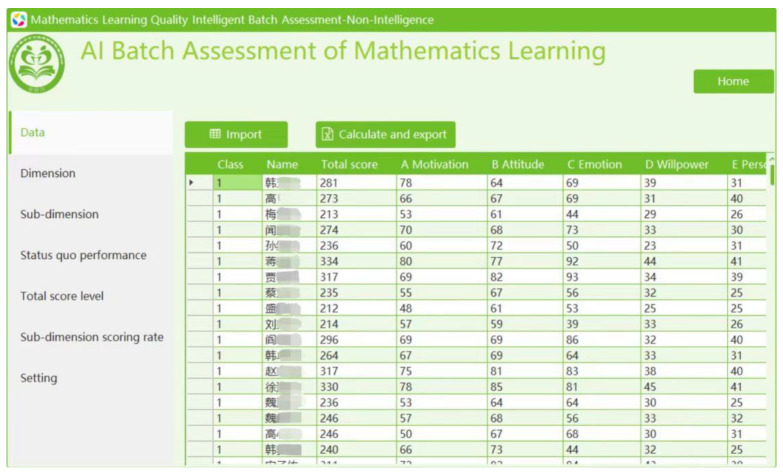
Batch assessment and strategy implementation system.

1. The individual self-assessment version. Students need to select their answers to the items in the questionnaire using the software. After providing answers to the items, they need to click “Next” until all the questions are answered. Then, they can click “Finish” to view their results (see Figure 5). The total score of the questionnaire, scores for the five dimensions, the norm-referenced ranks, and a radar chart comparing the scores of the principal dimensions with regional means are presented on the questionnaire results interface (Figure 6). Students can click “View Strategies” to find targeted strategies to improve their levels of non-intellectual factors (Figure 7).

2. The group assessment version. To illustrate, let us see how high school students’ levels of non-intellectual factors in mathematics are assessed on the system. Users can double-click to open the software and click “Import” to import an XLSX file titled “Student Math Non-intellectual Scores Table” into the system (see Figure 8). They can then view the dimensions, sub-dimensions, total score level, sub-dimension scoring rate, performance, and improvement strategy forms. When users click “Calculate and export”, the software will automatically export the Math Non-intellectual Factors Assessment Report (with enhancement strategies) for each student by school and class to the default folder.

The Intelligent Assessment and Strategy Implementation System, with a self-assessment version for individuals and another version for group assessment, developed independently by our team, is a software solution characterized by its scientific approach, high efficiency, precise assessment capabilities, and personalized intervention strategies. It aims to address the current lack of tools for assessing non-intellectual factors in mathematics learning. Additionally, the system’s group-oriented intelligent evaluation feature allows for the large-scale generation of assessment reports, which reduces the need for human and material resources. The personalized strategy implementation system, in turn, helps enhance students’ learning efficiency in mathematics.

### 3.2. Application of the Intelligent Assessment and Strategy Implementation System

#### 3.2.1. Comparison of Traditional and Smart Assessments

Most traditional assessments rely on paper-and-pencil tests or online questionnaires, which require researchers to spend a significant amount of time collecting and analyzing data and preparing diagnostic reports. This task not only demands that researchers have strong data analysis skills but also consumes considerable manpower and resources, resulting in inefficiency. Furthermore, traditional diagnostic reports generally offer broad improvement strategies based on assessment results, which often lack specificity.

**Figure 9:**
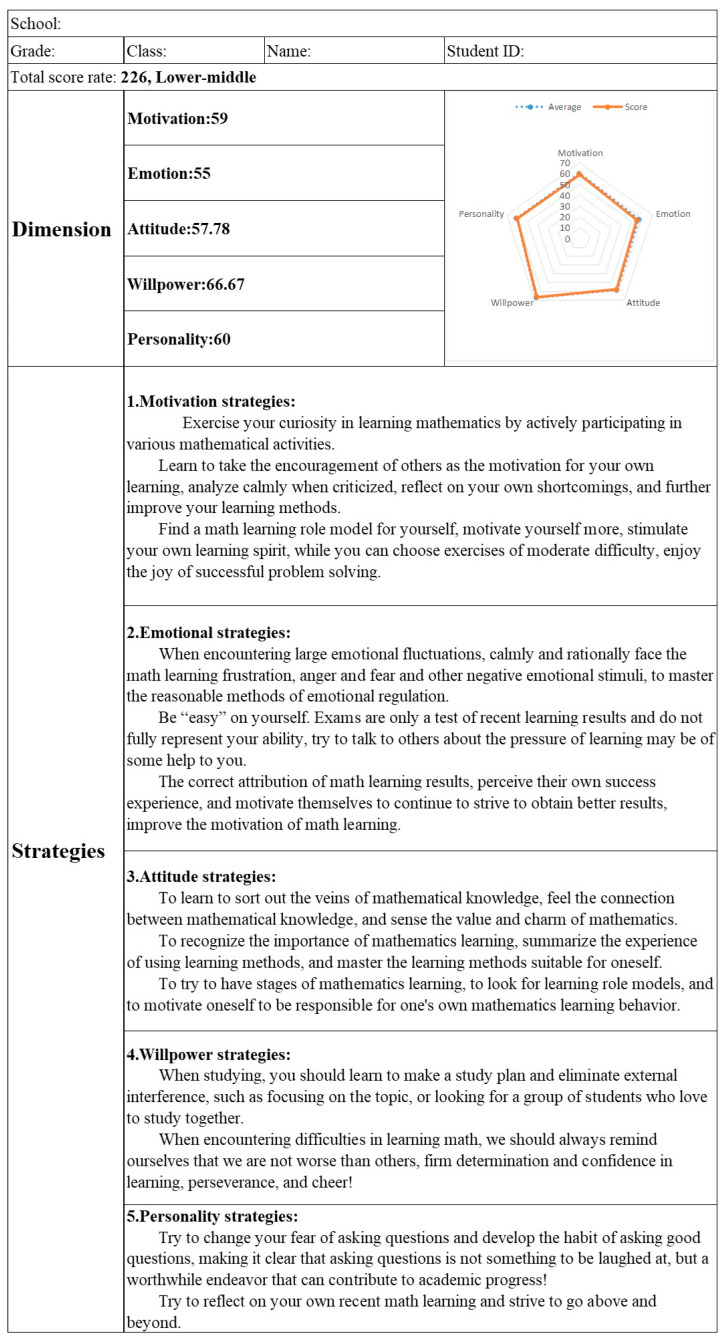
Diagnostic report on the lower-middle level of mathematics non-intellectual factors in a year-one senior high school student at a school in China.

The Intelligent Assessment and Strategy Implementation System developed in this study can intelligently analyze student questionnaire data. Within just 2 seconds, it can automatically generate 200 diagnostic reports at multiple levels (school-grade-class-individual), including total scores, dimension-specific scores, grade levels, radar charts comparing individual results to the mean, and precise, personalized improvement strategies (Figure 9). This system facilitates efficient diagnostic assessment and provides timely feedback on results and suggestions for improvement, thereby enhancing the efficiency, accuracy, and convenience of interventions aimed at improving the mathematical non-intellectual factors in senior high school students.

By comparing traditional assessments with the smart assessment developed in this study, evidence-based and empirical research methodologies were integrated to provide methodological support. In order to verify the effectiveness of the system, a combination of EBE and empirical research was adopted. On a national scale across China, the EBE method was used to analyze feedback from the experimental regions, information from the website of the Ministry of Education, and media reports. This provided extensive supportive evidence for the system’s application. In addition, case studies were conducted through empirical experiments in typical schools to validate the specific intervention effects of the system in real-world educational contexts. The combination of evidence-based and empirical approaches not only broadens the validation scope but also compensates for the limitations of using a single method, offering a comprehensive and scientific evaluation of the system’s effectiveness.

#### 3.2.2. Evidence-Based Effectiveness Testing of the Intelligent Assessment and Strategy Implementation System

Based on the principles of EBE, the Intelligent Assessment and Strategy Implementation System developed in this study has been widely implemented across various provinces and cities in China, garnering positive feedback. The education bureau of a district in City T remarked: “The system comprehensively diagnoses the non-intellectual factors affecting mathematics learning among elementary and secondary school students and provides precise, personalized improvement strategies. This has been a valuable exploration and contribution to the high-quality development of basic mathematics education in our district”. The Education and Sports Bureau of a county in BZ City, Province S, noted: “By utilizing intelligent technology, the system offers targeted improvement strategies based on students’ individual strengths and weaknesses in non-intellectual factors related to mathematics learning. This has significantly contributed to reducing the burden and improving the quality of mathematics education and teaching in our county, promoting the high-quality development of basic mathematics education”. The Institute of Educational Science in City C also provided feedback, stating: “The assessment project is scientifically advanced, with meticulously organized testing and precise data processing. The feedback reports from schools are accurate and scientifically grounded, and the improvement strategies are highly targeted and actionable. The system holds substantial instructional significance and practical value for improving high school students’ non-intellectual factors related to mathematics learning in our district”. The education bureau of a county in Province L commented: “Through the establishment of a localized norm for mathematics non-intellectual factors, we have been able to accurately assess the non-intellectual characteristics of our high school students’ mathematics learning. By conducting targeted interventions, the system has successfully enhanced these non-intellectual factors, improving the overall efficiency of mathematics teaching and learning. This has achieved the dual goal of reducing the academic burden and enhancing the quality of mathematics education”. The People’s Government of a county in Province Q gave the following praise: “Professor Wang’s team has developed a scientifically robust and reasonable tool for assessing non-intellectual characteristics in mathematics learning. The data analysis was conducted in a rigorous and standardized manner, and the resulting countermeasures and suggestions were highly targeted”.

Meanwhile, media reports have also validated the significant effectiveness of this system. According to the official website of the Ministry of Education of the People’s Republic of China (2021), this intelligent assessment system can promptly generate diagnostic reports; offer targeted improvement recommendations; and conduct large-scale and batch intelligent diagnoses at the regional, school, and individual student levels. The promotion results—obtained from more than 140 schools nationwide—demonstrate that the system effectively reduces the burden, improves the quality of mathematics learning, and offers valuable insights for reducing the burden in other subjects with the aid of AI technology. China Education News (2021) reported: “This intelligent batch assessment system can conduct large-scale, batch intelligent diagnoses at the regional, school, and individual student levels, automatically and efficiently producing diagnostic reports. Based on the identified issues, it automatically outputs targeted improvement strategies and delivers highly efficient, comprehensive, and personalized intelligent diagnostic services for regions, schools, and individual students. Simultaneously, it plays a comprehensive role in guiding, diagnosing, improving, and regulating assessments, facilitating the intelligent management of the quality of mathematics learning for primary and secondary school students”. China Information Weekly (Wang and Wu 2022) reported: “The intelligent assessment system has been employed to conduct mathematics learning quality improvement initiatives in over 600 schools across City B, City T, Province Q, Province Z, Province F, Province G, Province S, Province H, and Province X. It has achieved initial results in reducing the burden and enhancing the quality of mathematics learning. It has also accumulated valuable experience in alleviating the learning burden and improving the quality of learning in other subjects”.

In summary, an analysis of feedback from experimental districts, media reports (such as those from China Education News and China Information Weekly), and information from the Ministry of Education’s official website confirms that the system has significant capabilities for efficient diagnosis and precise strategy formulation. It provides students with scientific and efficient diagnostics for non-intellectual factors related to mathematics learning and helps students at different levels of these factors enhance their mathematics learning abilities through precise, personalized improvement strategies. The system has demonstrated its practical and research value in the field of education and has attracted widespread recognition and praise.

#### 3.2.3. Empirical Effects of Typical Cases Using the Intelligent Assessment and Strategy Implementation System

After the macro-level evidence-based research provided feedback on the overall effectiveness of the system in the experimental districts, a further empirical analysis was conducted at the micro-level, focusing on the specific practices of typical schools and students. Through a combination of feedback from the experimental districts and empirical data from typical cases in schools, a dual-perspective analysis was performed to deeply examine the system’s impact on enhancing non-intellectual factors in senior high school students in mathematics learning. In this part, with medium and lower-intermediate levels of mathematics non-intellectual factors, two 11th-grade classes at X School in City T were selected as experimental groups. A three-month intervention and improvement study was conducted based on the improvement strategies from the diagnostic reports. Meanwhile, another class with medium levels of mathematics non-intellectual factors was selected as the control group, which did not receive any intervention. The effectiveness of the Intelligent Assessment and Strategy Implementation System was evaluated through a quantitative analysis of improvements in mathematics and non-intellectual factors and a qualitative analysis of interview data and other materials.

1. Selection of Research Subjects

This study primarily focuses on improving and enhancing non-intellectual factors. Consequently, we selected classes with middle and below-middle performance for intervention. Before the intervention and improvement, all 11th-grade classes at X School in City T were evaluated on their mathematics non-intellectual factors levels, with reference to the regional norm for mathematics non-intellectual factors in senior high school students in City T (Wang et al. 2017) (Table 5). Following the assessment, we conducted a differential analysis on several middle-level classes to ensure the experimental and control groups were representative and comparable. To further bolster the scientific validity and practical feasibility of our subject selection, we collaborated with the school’s mathematics teaching and research group leader and grade-level leader. As a result, we designated Class 2 (middle level in mathematics non-intellectual factors) and Class 11 (below-middle level) as the experimental groups, with Class 12 (middle level) serving as the control group. This experimental design enables us to evaluate two key aspects: (1) Whether there is a significant improvement in mathematical non-intellectual factors among middle-level classes with and without intervention. (2) If there are notable differences in the enhancement of mathematical non-intellectual factors between below-middle and middle-level classes post-intervention.

2. Design of the Research Process

**Figure 10:**
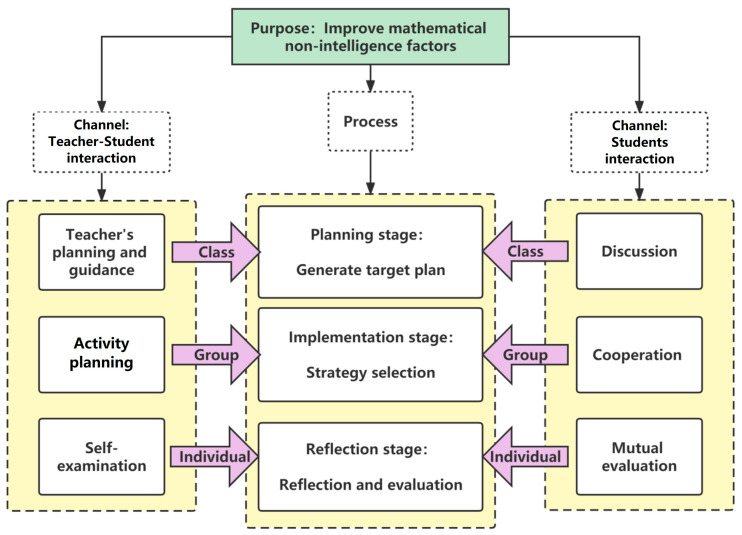
Flowchart of intervention and improvement.

The intervention process is based on Professor Zimmerman and Campillo’s (2003) three-phase cyclic model of self-regulated learning, focusing on the five dimensions of non-intellectual factors in mathematics learning. It explores the framework and design of interventions targeting non-intellectual factors in mathematics learning through teacher–student and student–student interactions, and it treats classroom, small-group, and individual interventions as the intervention targets. For each phase—planning, execution, and reflection—specific tasks were designed to guide teachers and facilitate students’ internalization of self-regulation. The overall intervention framework is illustrated in Figure 10.

3. Implementation of Intervention and Improvement

Intervention and improvement were implemented over a three-month period. The entire process was divided into six phases, corresponding to the five dimensions of non-intellectual factors in mathematics learning. Within each phase, the intervention followed a cyclical process of planning, execution, and reflection. The detailed implementation process is outlined in Table 6.

### 3.3. Effectiveness Testing of Intervention and Improvement

(1) Quantitative Analysis of Effectiveness

With the assistance of the researcher, a three-month intervention based on the improvement strategies suggested in the diagnostic report was conducted in the two experimental groups. After the intervention, the changes in the non-intellectual factors related to mathematics were measured for students in Experimental Group 1 (Class II), Experimental Group 2 (Class XI), and the Control Group (Class XII, which did not undergo the intervention). As shown in Table 7, Experimental Group 1 consisted of 43 students, 35 of whom (81.39%) showed improvements in their mathematics non-intellectual factors after the intervention, while 8 students (18.60%) showed no improvement. In Experimental Group 2, which consisted of 40 students, 32 (80.00%) improved, while 8 (20.00%) showed no improvement. In the Control Group of 44 students, only 9 (20.45%) improved without the intervention, and 35 (79.55%) showed no improvement. After the intervention, 80% or more of the students in the experimental groups showed improvement in their non-intellectual factors in mathematics. In contrast, approximately 80% of students in the control group showed no improvement. In conclusion, the Intelligent Assessment and Strategy Implementation System for Mathematics Non-intellectual Factors effectively enhanced students’ mathematics non-intellectual factors.

(2) Qualitative Analysis of Effectiveness

Following the intervention, interviews were first conducted with classroom teachers, mathematics teachers, and some students in Experimental Group 1 and Experimental Group 2. The interview content was organized and summarized, and excerpts from some students’ and teachers’ reflections on the intervention were analyzed. To protect confidentiality, pseudonyms were used for all interviewees.

① Student Interviews

<1> Motivation Dimension

Student A: The class activities were very refreshing, especially since they were combined with mathematics, which made me feel that math is not as dull as I thought—it can actually be interesting.

<2> Emotion Dimension

Student B: Through the “Strengths Bombing” activity, I discovered strengths in my math learning that I had not noticed before, which helped me build confidence in learning mathematics.

<3> Attitude Dimension

Student C: By learning about the applications and value of mathematics, I gained insights into math-related fields and professions, which sparked my strong interest in this area. During the “Math Learning Strategies Exchange Conference”, I found several math learning methods that work for me, which was very rewarding.

<4> Willpower Dimension

Student D: Every week, I now set a math study plan for the week. Since I have more self-directed time on weekends and might slack off, I make sure to schedule my homework for weekends, and if I complete it on time, I reward myself.

<5> Personality Dimension

Student E: My critical thinking and competitiveness have both increased. I found some errors in the after-class exercise answers and verified the results by discussing them with my classmates. I also signed up for a math competition. It was the first time I took the initiative to participate, even though I did not qualify for the next round.

② Teacher Interviews

<1> Class Teacher: After the class intervention, I noticed that the students’ enthusiasm for learning had increased. Many students now have clear learning goals, can develop study plans, and complete tasks efficiently and on time. The intervention not only improved their non-intellectual factors in mathematics learning but also positively impacted their attitudes toward other subjects.

<2> Mathematics Teacher: I found that the students’ interest in learning mathematics has increased through participating in the classroom intervention. Each student has developed their own approach to learning math, and they have successfully identified methods that suit their individual needs, which they are now able to integrate effectively into their math learning. Some students were able to critically question the content of the textbook, the answers to exercises, and the “small traps” set by the teacher in class. Their spirit of inquiry has significantly improved.

**Figure 11:**
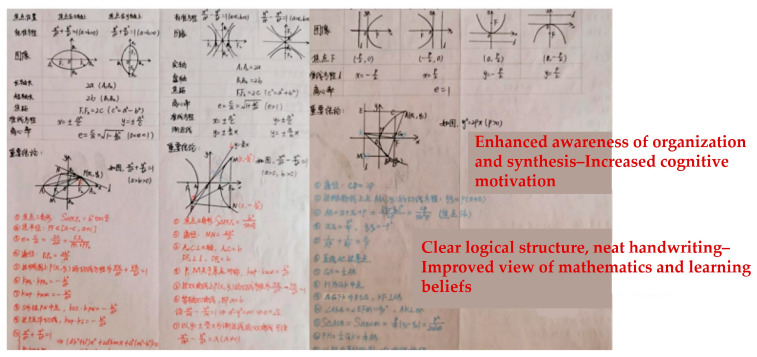
Math notes from a student in Experimental Group 1.

**Figure 12:**
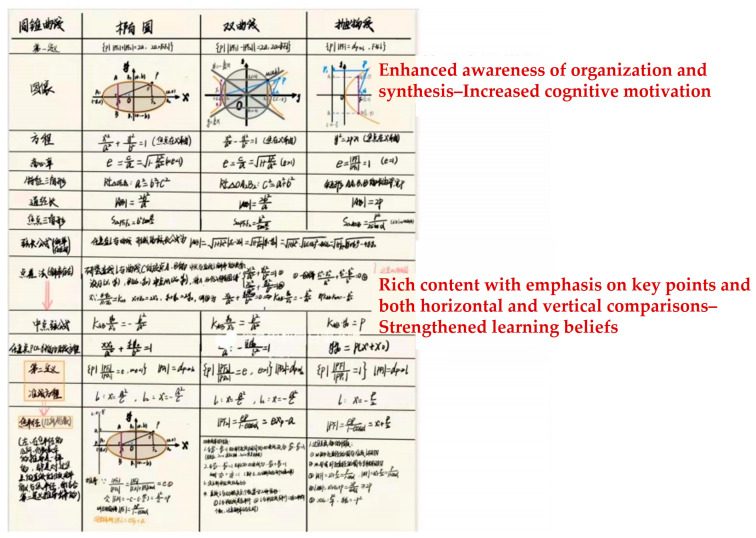
Math notes from a student in Experimental Group 2.

Second, students’ math notes and error correction logs were selected for analysis to provide evidence of the effectiveness of the intervention and improvement. For example:

During the non-intellectual intervention to improve math learning, students studied a unit on conic sections, including ellipses, hyperbolas, and parabolas. After the intervention, students actively summarized the material. Figure 11 and Figure 12 show notes from two students. These notes are logically structured, neatly organized, well-written, and comprehensive, and highlight key points, enhancing their understanding and retention of the different curves through longitudinal comparisons. This demonstrates that the intervention significantly increased students’ learning initiative, improved attitudes towards mathematics, and fostered effective learning methods, thereby enhancing non-intellectual factors in mathematics learning.

In summary, this study confirmed the significant effectiveness of The Intelligent Assessment and Strategy Implementation System in improving students’ non-intellectual factors related to mathematics through an intervention targeting year-two senior high school students at X Secondary School in City T. The results of the quantitative analysis and qualitative data indicated that students in the experimental classes showed substantial improvements in the dimensions of motivation, emotion, attitude, willpower, and personality. Their learning motivation and effectiveness were enhanced, and teachers reported a positive shift in the overall learning status and attitude of the students. In addition, this improvement was transferable to their performance in other subjects. This demonstrates the system’s capacity for delivering precise and personalized interventions.

## 4. Discussion and Conclusions

### 4.1. The Intelligent Assessment and Strategy Implementation System Can Efficiently and Comprehensively Diagnose Senior High School Students’ Mathematics Non-Intellectual Factors

Based on the assessment index system, scales, regional norms, and improvement strategies developed by our team, the Intelligent Assessment and Strategy Implementation System of Non-intellectual Factors in Mathematics designed in this study can efficiently and comprehensively diagnose students’ non-intellectual factors in mathematics. The rapid advancement of intelligent technologies is driving revolutionary changes in the field of educational assessment (González-Calatayud et al. 2021). Traditional assessments of non-intellectual factors are labor-intensive, time-consuming, and prone to errors (Simmreing et al. 2019). In contrast, the intelligent system developed in this study offers an innovative tool for educational assessment, enabling efficient diagnoses powered by big data. Through big data analysis, this intelligent system can accurately perform complex diagnostic tasks in a short amount of time. This efficiency applies not only to individual student assessments but also to large-scale evaluations of non-intellectual factors, effectively avoiding the inefficiencies and subjective biases associated with manual assessments and showcasing the system’s intelligent advantages. Research has consistently shown that factors such as motivation, emotion, attitude, willpower, and personality significantly influence students’ academic performance (Zhou and Wang 2024; Quílez-Robres et al. 2021; Sanchez-Ruiz and El Khoury 2019). By evaluating students across these five dimensions (motivation, emotion, attitude, willpower, and personality), the system comprehensively diagnoses their strengths and weaknesses in non-intellectual factors in mathematics. This approach transcends the traditional reliance on academic performance alone, providing a more scientific and comprehensive understanding of students’ learning behaviors from a non-intellectual perspective. Teachers can thus offer educational interventions tailored to each student’s individual needs.

In conclusion, the Intelligent Assessment and Strategy System developed in this study not only efficiently diagnoses mathematics non-intellectual factors but also provides a scientific foundation for students to better understand their strengths and weaknesses in non-intellectual aspects of mathematics learning. It likewise offers teachers a comprehensive view of their students’ non-intellectual strengths and shortcomings in mathematics, laying a scientific basis for informed interventions.

### 4.2. The Intelligent Assessment and Strategy Implementation System Provides Precise Strategy Support for Enhancing Non-Intellectual Factors in Mathematics Learning Among Senior High School Students

The Intelligent Assessment and Strategy Implementation System developed in this study not only efficiently diagnoses students’ mathematics non-intellectual factors but also provides precise, personalized improvement strategies to address deficiencies in the five dimensions of motivation, emotion, attitude, willpower, and personality. Research has demonstrated that personalized educational interventions, as one of the core principles of modern education, are an effective means of improving student learning outcomes (Zhou et al. 2022). Through the system’s precise policy-making feature, teachers can move beyond the traditional “one-size-fits-all” teaching approach and adopt strategies tailored to the unique needs of each student, effectively applying the principle of differentiated instruction. The evidence-based findings of this study, along with empirical case studies, confirm that the targeted and effective interventions provided by this intelligent system not only improve students’ non-intellectual factors in mathematics but also elevate their overall mathematics learning outcomes. This has earned the system widespread recognition from both students and teachers. Furthermore, the system’s precise interventions can create a ripple effect, positively influencing learning outcomes in other subjects, such as science. The evidence-based results of this study indicate that improvements in non-intellectual factors in mathematics have a cross-disciplinary impact. This is consistent with the findings of Nakakoji and Wilson (2020). The improvement strategies generated by this intelligent system are both highly actionable and effective, enabling students to enhance their mathematics non-intellectual factors and learning outcomes comprehensively. Through positive psychological mechanisms, these improvements transfer to other subjects, fostering a virtuous cycle of academic growth.

In summary, the Intelligent Assessment and Strategy System developed in this study enables precise interventions to improve students’ non-intellectual factors in mathematics learning, such as motivation, emotion, attitude, willpower, and personality.

Taken together, the Intelligent Assessment and Strategy Implementation System developed in this study features advanced visualization and intelligent capabilities, allowing it to efficiently and accurately diagnose students’ current levels of mathematics non-intellectual factors. The system’s personalized, intelligent strategy module provides targeted improvement strategies for addressing students’ deficiencies, facilitating precise educational interventions. Nevertheless, the system has certain limitations, such as the lack of integration of contextual factors, which prevents it from fully leveraging the advantages of big data in intelligent diagnosis and from deeply understanding the underlying causes of deficiencies in non-intellectual factors. In the future, the integration of contextual factors will be considered to further improve the Intelligent Assessment and Strategy Implementation System for Non-intellectual Factors in Mathematics Learning. The improved system will be used to thoroughly analyze the interactions between contextual factors and non-intellectual factors to identify the issues and underlying causes associated with non-intellectual factors in mathematics learning. Moreover, the system will be employed to conduct extensive applied research, ultimately enhancing the non-intellectual factors in senior high school students in mathematics learning more effectively. Moreover, there is an urgent need to expand the development of intelligent systems to encompass learning quality elements across other academic disciplines. For instance, we have already developed a preliminary version of an intelligent assessment system for scientific literacy and conducted a large-scale sample test in Tianjin, China, which yielded promising results. At the same time, our team is actively conducting research and development of new projects, such as an intelligent assessment system for the learning efficiency of primary and secondary school students. The corresponding applied research is expected to promote a comprehensive improvement in students’ learning effectiveness in mathematics.

## Figures and Tables

**Table 1:** Reliability indicators for the questionnaire data.

Dimension	Cronbach’s Alpha Coefficient	Split-Half Reliability	Test-Retest Reliability
Non-intellectual factors in mathematics	0.940	0.955	0.857
Motivation	0.904	0.791	0.805
Emotion	0.900	0.791	0.788
Attitude	0.864	0.865	0.812
Willpower	0.802	0.848	0.794
Personality	0.737	0.764	0.794

**Table 2:** Validity indicators of the questionnaire data.

Dimension	Non-Intellectual Factors in Mathematics	Motivation	Emotion	Attitude	Willpower	Personality
Non-intellectual factors in mathematics	1					
Motivation	0.868 ***	1				
Emotion	0.773 ***	0.594 ***	1			
Attitude	0.842 ***	0.683 ***	0.705 ***	1		
Willpower	0.753 ***	0.687 ***	0.502 ***	0.746 ***	1	
Personality	0.776 ***	0.587 ***	0.506 ***	0.535 ***	0.535 ***	11

Note: *** represents significant levels at 1%. The questionnaire has a solid theoretical foundation with a reasonable structural framework and good reliability and validity. Thus, it can be used as an effective instrument to measure the levels of non-intellectual factors in mathematics learning among high school students.

**Table 3:** The coding for the different dimensions.

Principal Dimension	Sub-Dimension	Number of Questions
Motivation-A (100 points)	Cognitive Motivation-A1 (45 points)	9
	External Motivation-A2 (20 points)	4
	Achievement Required-A3 (35 points)	7
Emotion-B (100 points)	Emotional Stability-B1 (25 points)	5
	Learning Anxiety-B2 (40 points)	8
	Learning Self-Efficacy-B3 (35 points)	7
Attitude-C (90 points)	View of Mathematics-C1 (35 points)	7
	Learning Beliefs-C2 (25 points)	5
	Learning Responsibility-C3 (30 points)	6
Willpower-D (45 points)	Self-discipline-D1 (25 points)	5
	Perseverance-D2 (20 points)	4
Personality-E (50 points)	Query Spirit-E1 (30 points)	6
	Competitiveness-E2 (20 points)	4

**Table 4:** Coding for performance and suggestions.

Dimension		Performance	Suggestion
Motivation-A (100 points)	Cognitive Motivation-A1 (45 points)	High level (X > 35): H-A1	S-A1
		Medium level (29 ≤ X ≤ 35): M-A1 Low level (X < 2 9): L-A1	S-A2
	External Motivation-A2 (20 points)	High level (X > 14): H-A2	S-A3
		Medium level (10 ≤ X ≤ 14): M-A2 Low level (X < 10): L-A2	S-A4
			S-A5
	Achievement Requires-A3 (35 points)	High level (X > 29): H-A3	S-A6
		Medium level (24 ≤ X ≤ 29): M-A3 Low level (X < 24): L-A3	S-A7
Emotion-B (100 points)	Emotional Stability-B1 (25 points)	High level (X > 22): H-B1	S-B1
		Medium level (17 ≤ X ≤ 22): M-B1 Low level (X < 17): L-B1	S-B2
	Learning Anxiety-B2 (40 points)	High level (X > 32): H-B2	S-B3
		Medium level (26 ≤ X ≤ 32): M-B2 Low level (X < 26): L-B2	S-B4
	Learning Self-Efficacy-B3 (35 points)	High level (X > 26): H-B3	S-B5
		Medium level (21 ≤ X ≤ 26): M-B3 Low level (X < 21): L-B3	S-B6
Attitude-C (90 points)	View of Mathematics-C1 (35 points)	High level (X > 29): H-C1	S-C1
		Medium level (23 ≤ X ≤ 29): M-C1 Low level (X < 23): L-C1	S-C2
	Learning Beliefs-C2 (25 points)	High level (X > 22): H-C2	S-C3
		Medium level (18 ≤ X ≤ 22): M-C2 Low level (X < 18): L-C2	S-C4
	Learning Responsibility-C3 (30 points)	High level (X > 26): H-C3	S-C5
		Medium level (22 ≤ X ≤ 26): M-C3 Low level (X < 22): L-C3	S-C6
Willpower-D (45 points)	Self-discipline-D1 (25 points)	High level (X > 21): H-D1	S-D1
		Medium level (17 ≤ X ≤ 21): M-D1 Low level (X < 17): L-D1	S-D2
	Perseverance-D2 (20 points)	High level (X > 17): H-D2	S-D3
		Medium level (13 ≤ X ≤ 17): M-D2 Low level (X < 13): L-D2	S-D4
Personality-E (50 points)	Query Spirit-E1 (30 points)	High level (X > 22): H-E1	S-E1
		Medium level (19 ≤ X ≤ 22): M-E1 Low level (X < 19): L-E1	S-E2
	Competitiveness-E2 (20 points)	High level (X > 14): H-E2	S-E3
		Medium level (12 ≤ X ≤ 14): M-E2 Low level (X < 12): L-E2	S-E4

**Table 5:** Descriptive statistics of overall levels of non-intellectual factors in mathematics for senior high school classes.

Class	Number of Cases	Mean Score	Level	Rankings
Class I	34	272.71	Middle	1
Class II	43	253.44	Middle	10
Class III	39	264.75	Middle	6
Class IV	26	263.04	Middle	7
Class V	37	262.11	Middle	9
Class VI	40	262.81	Middle	8
Class VII	41	271.21	Middle	3
Class VIII	38	269.69	Middle	4
Class IX	42	272.60	Middle	2
Class X	34	264.95	Middle	5
Class XI	40	223.78	Lower-middle	12
Class XII	44	249.86	Middle	11

**Table 6:** Elements of intervention and improvement.

Intervention Content	Intervention Stages	Teacher–Student Interaction	Student–Student Interaction
Intervention 1—Recognizing non-intellectual factors in mathematics learning	Planning stage	(1) Teachers set intervention goals. (2) Teachers design ice-breaking activities (e.g., Stickman Rush, Intelligent Roll Call) to stimulate group dynamics.	Students discuss and negotiate to set goals.
		Finalize the objectives: (1) Understand the components of non-intellectual factors in mathematics learning based on the information provided by the teacher. (2) Analyze personal challenges in mathematics learning based on these components and the personal diagnostic report.	
	Implementation stage	Activity 1: Case Study Presentation—The Pity of Zhong Yong Activity 2: Teacher-Led Inquiry	Activity 1: Read materials provided by the teacher to understand the non-intellectual factors in mathematics learning. Activity 2: Students share their views and analyze the task.
	Reflection stage	Activity: Complete the Feedback Form on Intervention and Improvements for Non-intellectual Factors in Mathematics Learning.	Activity: Student Self-Evaluation Time
Intervention 2—Motivational dimension: fun math, happy learning	Planning stage	Teachers set intervention goals.	Students discuss and negotiate to set goals.
		Finalize the objectives: (1) Search for mathematics-related books (including the history of mathematics, fun mathematical puzzles, mathematical novels, etc.), gain a basic understanding of the content, and choose sections to read during extracurricular time to enhance interest in mathematics and understanding of the subject. (2) Learn about world-renowned mathematicians and their outstanding achievements and explore the connections between their work and what has been learned to stimulate curiosity and enhance the need for achievement in mathematics learning.	
	Implementation stage	Activity 1: Möbius Strip Activity 2: Drawing Ellipses with Origami	Strategy Options: S-A2, S-A7 (Table 4)
			Activity 1: What Do You Know About Mathematicians? Activity 2: Setting Mathematics Learning Goals
	Reflection stage	Review the key points of the lesson and re-emphasize the important role of motivation in effective mathematics learning and personal growth.	
Intervention 3—Emotional dimension: I am in charge of my emotions	Planning stage	Teachers set intervention goals.	Students discuss and negotiate to set goals.
		Finalize the objectives: (1) Reflect on past mathematics learning experiences, build confidence through memorable successes, and relive the successful experience of learning mathematics. (2) Apply the theory of attributing success and failure by reflecting on gains and losses in the midterm mathematics exam. (3) Share mathematics learning experiences, learn from each other, explore personalized learning strategies, and identify new breakthroughs.	
	Implementation stage	Activity 1: Strengths Savings Bank Activity 2: Attribution Training Activity 3: Mathematics Learning Strategies Exchange Conference	Strategy Options: S-B5, S-B7 (Table 4)
			Activity 1: Strengths Bombardment Activity 2: Attribution Training for Midterm Exam Success or Failure
	Reflection stage	Review the key points of the lesson and re-emphasize the important role of emotions in mathematics learning and their impact on learning efficiency and personal growth.	
Intervention 4—Attitude dimension: attitude determines altitude, and details determine success	Planning stage	Teachers set intervention goals.	Students discuss and negotiate to set goals.
		Finalize the objectives: Construct connections between different chapters of mathematical knowledge (e.g., ellipse, hyperbola, and parabola in the conic sections chapter), begin creating a mind map, and form a network of mathematical knowledge.	
	Implementation stage	Activity 1: The Nature of Mathematics Activity 2: The Value of Mathematics	Strategy Selection: S-C2 (Table 4)
			Activity: Create a Mathematical Knowledge Mind Map
	Reflection stage	Review the key points of the lesson and re-emphasize the role of attitudes in enhancing the efficiency of mathematics learning and fostering personal growth.	
Intervention 5—Willpower dimension: giving up is easy, but perseverance is cool	Planning stage	Teachers set intervention goals.	Students discuss and negotiate to set goals.
		Finalize the objectives: (1) Understand that willpower is an important factor in improving the effectiveness of mathematics learning. (2) Select role models in mathematics learning. (3) Develop a mathematics study plan.	
	Implementation stage	Activity: Explore the Lives of Mathematicians	Strategy Selection: S-D2 (Table 4)
			Activity 1: The Power of Role Models Activity 2: Develop a Small Plan
	Reflection stage	Review the key points of the lesson and re-emphasize the importance of willpower in enhancing the efficiency of mathematics learning and personal growth.	
Intervention 6—Personality dimension: challenging authority, and challenging self	Planning stage	Teachers set intervention goals.	Students discuss and negotiate to set goals.
		Finalize the objectives: Organize competitive activities and encourage class members to participate in groups.	
	Implementation stage	Activity: Use mathematics learning as an example to discuss perspectives on “involution”.	Strategy Selection: S-E5 (Table 4)
			Activity 1: Class Math Knowledge Quiz Activity 2: Math Modeling Simulation
	Reflection stage	Review the key points of the lesson and re-emphasize the importance of personality in enhancing the efficiency of mathematics learning and personal growth.	

**Table 7:** Improvement of non-intellectual factors in mathematics in Experimental Group 1, Experimental Group 2, and Control Group through intervention.

Groups	N	Effect	Number	Percentage
Experimental group 1	43	Improved	35	81.39%
		Not improved (including unchanged)	8	18.61%
Experimental group 2	40	Improved	32	80.00%
		Not improved (including unchanged)	8	20.00%
Control group	44	Improved	9	20.45%
		Not improved (including unchanged)	35	79.55%

## Data Availability

The data presented in this study are available on request from the corresponding author. The data are not publicly available due to privacy.

## Supplementary Materials

The following supporting information can be downloaded at: https://www.mdpi.com/article/10.3390/jintelligence12120126/s1, Supplementary File S1: Suggestions for Enhancing Non-Intelligence Factors in Mathematics Achievements—Teacher’s Edition; Supplementary File S2: Enhancement Strategies for “Non-Intellectual Factors among Senior High School Students”—For Students.
