# Correction: Kang et al. (2024). The Development and Application of an Intelligent Assessment and Strategy Implementation System for Non-Intellectual Factors in Mathematics Learning Among Senior High School Students. *Journal of Intelligence* 12: 126

**DOI:** 10.3390/jintelligence13030024

**Published:** 2025-02-20

**Authors:** Yueyuan Kang, Guangming Wang, Luxuan Liu, Jing Liu, Qianqian Gao

**Affiliations:** 1 Faculty of Education, Tianjin Normal University, Tianjin 300387, China; 2 Tianjin Academy of Educational Science, Tianjin 300191, China

## Additional Affiliation

In the published publication (Kang et al. 2024), there was an error regarding the affiliation for Guangming Wang. Change of affiliation from “1” to “2”, the updated affiliation should include “Tianjin Academy of Educational Science, Tianjin 300191, China”. The authors state that the scientific conclusions are unaffected. This correction was approved by the Academic Editor. The original publication has also been updated.

## References

[B1-jintelligence-13-00024] Kang Yueyuan, Wang Guangming, Liu Luxuan, Liu Jing, Gao Qianqian (2024). The Development and Application of an Intelligent Assessment and Strategy Implementation System for Non-Intellectual Factors in Mathematics Learning Among Senior High School Students. Journal of Intelligence.

