# Supplementary material for: The Development and Application of an Intelligent Assessment and Strategy Implementation System for Non-Intellectual Factors in Mathematics Learning Among Senior High School Students
Source: J Intell. 2024 Dec 11;12(12):126. doi: 10.3390/jintelligence12120126 (PMC11676365; doi:10.3390/jintelligence12120126)
Supplement: Supplementary file 1 [file jintelligence-12-00126-s001.zip › Supplementary Materials File S1 Suggestions for Enhancing Non-Intelligence Factors in Mathematics Achievements - Teacher's Edition.pdf]

Supplementary Materials File S1: Suggestions for Enhancing *Non-Intelligence Factors in Mathematics Achievements* - Teacher's Edition

Dimensions	Operational definitions	Suggestions
Motivation	**Cognitive motivation:** the reason, purpose, or impetus for a student to undertake mathematics learning; curiosity about mathematics; the tendency to learn and to acquire mathematical knowledge.	**1. Adding knowledge related to mathematics to enable students to fully realize the value of mathematics**<br>Introducing students to supplementary knowledge related to the content of this section (including mathematicians' achievements, historical sources of knowledge, process of mathematical development, outstanding contributions, applications, and famous historical problems, etc.). By understanding the historical sources of mathematics, students can recognize its cultural value. By learning about the outstanding contributions of mathematics, students can recognize its application value. Through feeling the scientific research spirit of mathematicians, students can recognize its educational value. Furthermore, through learning the process of solving famous historical problems, students can recognize the scientific value of mathematics.<br>**2. Organizing a variety of mathematical activities to stimulate students' interest in learning**<br>Organizing various forms of mathematical activities, such as quizzes, modeling, exploratory activities, etc. and enriching the content and form of activities. By incorporating hands-on practice (e.g., cutting a circle, drawing an ellipse using a line, dividing an angle in three equal parts, etc.), independent inquiry, cooperative exchanges, etc. into activities, students can give full play to their subjectivity. Accordingly, such activities stimulate the interest in learning mathematics among students.<br>**3. Increasing emotional engagement to arouse students' interest in learning mathematics**<br>Increasing emotional engagement in mathematical teaching. In the classroom, teachers should refine their pedagogical language and choose their style according to their own teaching style. For example, teachers can use humorous and vivid language to attract students' attention. Outside the classroom,

		teachers should take the initiative to communicate with students, approaching and communicating with them through equal and cordial dialogues to establish a good relationship between them. With a proper degree of emotional engagement, teachers can better evoke students' interest in learning mathematics.<br>**4. Integrating real-life elements into mathematics teaching to cultivate students' interest in learning mathematics**<br>Using real-life teaching aids (i.e., using the boundaries of the blackboard to introduce line segments). Creating real-life problems (i.e., asking students to use the Pythagorean Theorem to calculate the height of a flagpole). Using mathematical language more often to describe real-life scenes. These measures and other similar methods can guide students to appreciate that mathematics is everywhere in real-life. Accordingly, teachers can prompt students to connect mathematics and life, thereby cultivating students' interest in learning mathematics.<br>**5. Enhancing students' interest in learning mathematics with the help of information technology and other modern teaching tools**<br>Using multimedia tools, geometry drawing board, and GGB software to present dynamic mathematical scenarios and increase students' interest in learning mathematics. For example, when teaching angles, GGB can be used to demonstrate the movement of rays, thereby helping students to learn the sides and size of an angle. Using multimedia tools to display news reports or historical sources related to the content being taught to increase students' interest in learning mathematics while studying interesting materials.<br>**6. Focusing on the diversification of mathematical exercises to enhance students' curiosity in learning mathematics**<br>Providing students with open-ended questions or topics for discussion with multiple solutions. Encourage students to practice multiple solutions, analyze and answer mathematical problems from different perspectives, and find different ways of

		solving them. Asking students to seek the simplest solutions in the world of mathematics to enhance their curiosity in learning the subject.
	**External motivation:** external forces that motivate and guide students to engage in learning mathematics.	**1. Expressing clear expectations for students to increase their motivation to learn**<br>Expressing clear expectations to students. For example, when teachers wish to complete a specific pedagogical task or requirement, they should clearly tell students what they are expected to do, how they will be evaluated, and what the rewards or penalties will be for successes and failures. When students are clear about what to work on and what results to expect, they are more motivated to learn mathematics.<br>**2. Adopting a multi-dimensional evaluation system to make objective and fair evaluations of students**<br>Combining process and summative assessment when giving evaluations to students. Refining the evaluation criteria when making process assessments. For example, when evaluating the performance of students in a specific class, teachers can give judgments based on multiple dimensions, such as whether they display a positive learning attitude, answer a question correctly, contribute to group cooperation, etc. Paying attention to the diversification of evaluating subjects when making summative assessment. For example, when evaluating students' daily performance at the end of a semester, teachers should integrate their own, parents', and student self-comments to derive a more comprehensive and objective evaluation.<br>**3. Giving more rewards and less punishment in the process of teaching to enhance students' motivation to learn mathematics**<br>When students answer questions or make progress in test scores, teachers should praise them or give them material rewards. When students are distracted in class or regress in test scores, teachers can talk to them to understand the reasons and further help them correct their mistakes. Even when punishments are necessary to let students bear the consequences of their behavior, teachers should take care not to do so in public and not to hurt students' self-esteem.
