# Supplementary material for: The Development and Application of an Intelligent Assessment and Strategy Implementation System for Non-Intellectual Factors in Mathematics Learning Among Senior High School Students
Source: J Intell. 2024 Dec 11;12(12):126. doi: 10.3390/jintelligence12120126 (PMC11676365; doi:10.3390/jintelligence12120126)
Supplement: Supplementary file 1 [file jintelligence-12-00126-s001.zip › Supplementary Materials File S2 Enhancement Strategies for 'Non-Intellectual Factors among Senior High School Students' - For Students.pdf]

Supplementary Materials File S2: Enhancement Strategies for *"Non-Intellectual Factors among Senior High School Students"* – For

Students

Note: The questionnaire has a maximum score of 385 points, with X representing the raw score.

Dimension		Performance	Suggestion
Motivation (100 points)	Cognitive motivation (45 points)	High-level students (X ≥ 32):<br>1. Fully recognize the value of learning mathematics.<br>2. Have a keen interest in learning mathematics.<br>3. Possess a strong curiosity to learn mathematics.	Well done! Keep it up.
		Intermediate-level students (27 ≤ X < 32):<br>1. Room for improvement exists in recognizing the value of learning mathematics.<br>2. Room for improvement exists in terms of interest in learning mathematics.<br>3. Room for improvement exists in the curiosity to learn mathematics.<br>Low-level students (X<27):<br>1. Perform poorly in recognizing the value of learning mathematics.<br>2. Perform poorly in terms of interest in learning math.<br>3. Perform poorly in the curiosity to learn	1. Broadening knowledge related to mathematics to fully recognize the value of the subject<br>Broaden mathematics-related knowledge according to the content of each session by reading relevant books, documents, or online materials. Such content mainly includes the achievements of mathematicians, and the origins, development, major contributions, applications, and famous problems of mathematical concepts. By understanding the origins of such concepts, you will recognize the cultural value of mathematics. By learning about the major contributions of the concepts, you will recognize the scientific value of mathematics. By understanding the

		mathematics.	scientific spirit of scientists, you will learn about the educational value of mathematics. By examining the solution of famous problems, you will realize the scientific value of math.<br>2. Actively participate in various math activities to stimulate interest in math learning<br>Actively participate in both in-class and after-class math activities, with full participation and engagement in the former and optional participation in after-class math activities of interest. Engage yourself in the activities through hands-on practice (e.g., cutting a circle, drawing an ellipse using a line, dividing an angle in three equal parts, etc.), independent investigation, and cooperation and communication. Be a proactive learner to stimulate interest in math learning.<br>3. Practice finding multiple solutions for individual math problems to enhance the curiosity in math learning<br>Practice finding multiple solutions for individual math problems by yourself or with other students to analyze and solve the problems from different perspectives, find different ways to solve problems, and seek the simplest solutions in the world of mathematics, ultimately to enhance curiosity to learn mathematics.

	External motivation (20 points)	High-level students (X ≥ 13):<br>1. Show weaknesses in their attitudes toward external incentives, particularly by placing too much importance on evaluations, rewards and punishments from teachers, parents, and peers. Susceptible to the influence of teachers' behaviors and test results.	Do not over-value rewards to avoid developing an excessively utilitarian mindset<br>Do not overvalue external rewards. For example, do not regard verbal or material rewards from teachers and parents as your goal for math learning. Instead, treat them as a means to motivate your own learning to avoid developing an overly utilitarian mindset.
		Intermediate-level students (10 ≤ X < 13):<br>1. Have excellent attitudes toward external incentives. Specifically, they remain objective in the evaluations, rewards and punishments from teachers, parents and peers. In addition, they are not susceptible to the influence of teachers' behaviors and test results.	Well done! Keep it up.
		Low-level students (X<10):<br>1. Perform poorly in their attitudes toward external incentives, particularly by caring nothing about the evaluations, rewards and punishments from teachers, parents and peers. In addition, they are not susceptible to the influence of teachers' behaviors and test results.	Treat external evaluations reasonably and adjust math learning behavior<br>To discern whether your teachers, parents, and peers are objective and fair in their evaluations is important. If their evaluations are objective and fair, you should consider the good ones, such as praises, as motivation for learning. For the negative ones, such as criticisms, you should analyze them calmly and reflect on your own math learning behaviors from the evaluations. This will help you compensate for your weaknesses

			and further improving your math learning behaviors. If their evaluations are neither objective nor fair, you can just ignore them.
	Achievement requires<br>(35 points)	High-level students ($X \geq 27$):<br>1. Have specific math learning goals.<br>2. Have a strong need to succeed and demonstrate their competence in math.	
		Intermediate-level students ($23 \leq X < 27$):<br>1. Room for improvement exists in setting specific math learning goals.<br>2. .Room for improvement exists in the need to succeed and demonstrate their competence in math.<br>Low-level students ($X<23$):<br>1. Perform poorly in setting specific math learning goals.<br>2. Perform poorly in terms of the need to succeed and demonstrate their competence in math.	1. Set reasonable and specific learning goals to stimulate motivation in math learning<br>Set specific learning goals for each session, month, or semester based on your math skills. These goals should practical and achievable through hard work. Keep them specific and detailed. You can list them one by one. Regularly update your learning goals as your math skills change to stimulate motivation for math learning.<br>2. Select exercises of moderate difficulty to experience the joy of problem solving<br>Select problems of moderate difficulty according to your current math skills, ultimately ensuring that these challenges can be successfully addressed if you make some efforts. After successfully solving a moderately challenging problem, you can experience and enjoy the satisfaction of the accomplishment.

			3. Act positively to take advantage of opportunities to enhance the need to demonstrate your competence in mathematics<br>Actively answer questions and propose your ideas and conjectures in class. Meanwhile, respond to the teacher's questions and the implementation of teaching content. During after-class hours, take the initiative to communicate with your classmates about content related to mathematics learning, such as exercises, activities, and learning experiences. Furthermore, give full play to your strengths in group work.
Emotion (100 points)	Emotional stability (25 points)	High-level students ($X \geq 21$) ：<br>1. Have a comprehensive understanding of the nature, characteristics, and types of emotions.<br>2. Can control their intense negative emotions.<br>3. Can regulate their intense negative emotions in a timely manner.	Well done! Keep it up.
		Intermediate-level ($17 \leq X < 21$):<br>1. Room for improvement exists in identifying their emotions.<br>2. Room for improvement exists in the control of intense emotions.<br>3. Room for improvement exists in the	1. Learn about emotions and enhance awareness of your own emotions<br>To understand that there are no good or bad emotions, and that emotions are generally categorized as positive and negative. However, good or bad behaviors that are caused by emotions exist, which result in good or bad

		regulation of negative emotions.<br>Low-level students (X<17):<br>1. Perform poorly in identifying their emotions.<br>2. Perform poorly in controlling their intense emotions.<br>3. Perform poorly in regulating negative emotions.	consequences. By learning about emotions, you will be able to understand that when you are dealing with your own such as anger and frustration, you need to manage these so that they will not cause undesirable behaviors.<br>2. Stay calm and rational when dealing with negative emotions, such as frustration, anger and fear, in mathematics learning, and effectively manage intense emotions<br>When encountering stronger emotional stimuli, stay rational and force yourself to calm down. Analyze the reasons before deciding to express your emotions or get your impulses under control, so as to effectively manage your intense emotions.<br>3. Acquire scientific emotional regulation skills and learn to effectively regulate intense negative feelings in math learning<br>Learning scientific and effective emotional regulation strategies, such as language suggestion techniques, goal shifting methods, environmental adjustment strategies, and self-expression techniques. By distracting yourself and changing an environment, you can shift your emotional focus and release intense emotions that occur during math learning, such as frustration, anger, and fear.

	Learning anxiety (40 points)	High-level students (X ≥ 32):<br>1. Do great jobs in leaning math and dealing with exercises, symbols, and equations.<br>2. Do great jobs in relieving math stress.	Well done! Keep it up.
		Intermediate-level students (26 ≤ X < 32):<br>1. Room for improvement exists in learning math and dealing with exercises, symbols, and equations.<br>2. Display average performance in relieving math stress.<br>Low-level students (X<26):<br>1. Exhibit weaknesses deficiencies in learning math and dealing with exercises, symbols, and equations.<br>2. Do great jobs in relieving math stress.	1. Be more tolerant toward yourself to reduce math learning anxiety<br>Be more tolerant toward yourself regarding math learning and math exams. Do not be too hard on yourself. Avoid the pursuit of perfection. Relax in times of difficulties and frustrations. Do not hurry for results, allow occasional mistakes in math learning, and gradually adjust yourself to the pace of math learning in senior high school.<br>2. Actively communicate with others about your learning experience and reduce math stress<br>Actively communicate with your teachers, parents, or peers about your math learning experience to reduce math stress. For example, you may be experiencing difficulty in focusing in class or finding effective learning methods. Ask others about their experience or seek suggestions to reduce such stress.<br>3. Properly understand math learning anxiety and turn it into motivation for math learning<br>Learn about anxiety and understand its role. Properly and scientifically understand math learning anxiety to

			recognize that an appropriate level can promote math learning and turn anxiety into learning motivation, thus reducing its impact.
	Learning self-efficacy (35 points)	High-level students ($X \geq 21$)：<br>1. Can make positive subjective judgments about their levels of mathematical skills and their ability to learn mathematics.<br>2. Highly motivated to learn math.<br>3. Have strong confidence in learning math.	1. Fully understand and recognize your level of math skills and mathematical learning ability through tests as well as teacher assessment, peer and self-assessment Comprehensively understand and recognize your level of math skills and mathematical learning ability from tests as well as from teacher, peer, and self-assessment. In addition, regularly update your understanding of your level of math skills and mathematical learning ability based on test scores and external evaluations.<br>2. Maintain and improve motivation for math learning by making correct attributions for the results of such learning<br>Understand that the outcome of mathematics learning is affected by various factors, including internal factors such as one's own ability and effort, and external factors such as learning difficulty and physical and mental state. When dealing with the outcomes, attribute bad outcomes, such as poor test results or unsatisfactory homework performance, to external factors or the internal factor of not working hard enough. Avoid attributing negative outcomes to
		Intermediate-level students ($21 \leq X < 26$):<br>1. Room for improvement exists in making positive subjective judgments and predictions about their levels of mathematical skills.<br>2. Room for improvement exists in making positive subjective judgments and predictions about their ability to learn mathematics.<br>3. Room for improvement still exists in their confidence in math learning.<br>Low-level students ($X<21$):<br>1. Display weaknesses in making positive subjective judgments and predictions about their levels of mathematical skills.<br>2. Display weaknesses in making positive subjective judgments and predictions about	

		their ability to learn mathematics.<br>3. Not confident in math learning.	inadequate ability, as such attribution will reduce your own motivation to learn mathematics. On the contrary, attribute good outcomes, such as satisfying test results and good homework performance, to your own ability and effort. Believe that you are a capable math learner and encourage yourself to keep working hard to achieve better results and improve your motivation to learn mathematics.<br>3. Perceive your successful experiences to boost your confidence in learning mathematics<br>Think about your successful experiences in answering questions in class and engaging in group investigation and other mathematical activities to positively perceive the rewards, the teacher's praise, and positive learning experience you receive in these activities. By doing so, you can see that you can learning mathematics well, thus boosting your confidence.
Attitude (90 points)	View of mathematics (35 points)	High-level students ($X \geq 28$):<br>1. Excel in their understanding and attitude towards mathematical knowledge.<br>2. Excel in their understanding and attitudes toward the value of math.	Well done! Keep it up.
		Intermediate-level students ($24 \leq X < 28$):<br>1. Perform moderately well in their	1. Fully understand the origin and development of mathematical knowledge to deepen the

		understanding and attitudes toward mathematical knowledge.<br>2. Room for improvement exists in the understanding and attitudes towards the value of math.<br>Low-level students (X<24):<br>1. Perform poorly in their understanding and attitudes toward mathematical knowledge.<br>2. Display weaknesses in their understanding and attitudes toward the value of mathematics.	comprehension and form a proper attitude toward mathematical knowledge<br>Learn about the origin and development of mathematical knowledge by reading or searching online to comprehend the historical and logical order of such knowledge and the differences between these two aspects. By doing so, you will deepen your understanding of mathematical knowledge and form a proper attitude toward mathematical knowledge.<br>2. Efficiently use mind maps to deeply understand the intrinsic connections between mathematical concepts<br>Use mind maps to portray the structures and connections within and between chapters. Differentiate these concepts and relationships with the help of different shapes and colors to gain a deep and clear understanding of the intrinsic connections between mathematical concepts.<br>3. Participate in various math learning activities to appreciate the value of mathematics<br>Understand the rigor and scientific nature of mathematics by learning about various concepts, establishing theorems, and solving mathematical problems. Apply mathematical knowledge to solve practical problems to understand the wide applications of mathematics. Read stories of mathematicians and books (non-textbook) on

			mathematics to understand the cultural charm of mathematics. Participate in moral education activities to understand the educational role of mathematics. For example, apply knowledge about rational numbers to calculate how many garbage bags are needed weekly at home, thus improving your environmental awareness and understanding the educational value of mathematics.
	Learning beliefs (25 points)	High-level students (X ≥ 21) :<br>1. Know how to learn mathematics effectively.<br>2. Have a good attitude toward math learning.	Well done! Keep it up.
		Intermediate-level students (18 ≤ X < 21):<br>1. Room for improvement in learning methods still exists.<br>2. Room for improvement still exists in their attitude toward math learning.<br>Low-level students (X<18):<br>1. Perform poorly in terms of learning methods.<br>2. Perform poorly in their attitudes toward math learning.	1. Summarize your experience of using various learning methods and find those that work for you Summarize your experience of using various learning methods, consider what is a good method for you, improve your learning methods in the process of learning mathematics, and finally find those that work for you. (Here, we would like to provide some good learning methods for your reference: use the textbook for effective before-class study, mark the key points and difficult points encountered, mark the places where you have questions, and listen to the lecture with questions; listen carefully and take effective notes in class; and review the lessons in time after class by

			reviewing your notes and creating mind maps.)<br>2. Recognize the importance of mathematics learning and develop a serious attitude toward such learning<br>Observe and discover real-life scenarios and think positively about whether the scenarios can be linked to the mathematical knowledge that you have learned. During the thinking process, you will realize that mathematics can be applied to life and recognize the importance of adopting a serious attitude toward math learning. This will help you remain determined in learning mathematics and develop a serious attitude toward such learning.<br>3. Build up confidence in math learning and cultivate a positive attitude toward learning<br>You should believe that you are talented, that you are a natural math learner, and that you can learn it well. Actively engage in such learning with confidence, such as by listening attentively to lectures and actively completing math homework, and develop a positive attitude toward learning.
	Learning responsibility (30 points)	High-level students ($X \geq 25$):<br>1. Active in math learning.<br>2. Take responsibility for their own behavior in learning mathematics.	Well done! Keep it up.

		Intermediate-level students (21 ≤ X < 25) ：<br>1. Room for improvement in active math learning exists.<br>2. Room for improvement exists in taking responsibility for their own behavior in learning mathematics.<br>Low-level students (X<21):<br>1. Perform poorly in active math learning.<br>2. Perform poorly in taking responsibility for their own behavior in learning mathematics.	1. Recognize that you are in control of your learning behavior and remind yourself to be an active math learner through self-suggestion<br>Understand that the outcome of learning depends largely on your own learning behaviors and further recognize that you are in control of such behavior, so that you should be active in learning math. Accordingly, you can remind yourself to be active in math learning verbally or mentally. For example, remind yourself to be proactive in completing your math homework by silently saying to yourself, "There's much homework to do this time, so I'm going to hurry up and get started on it."<br>2. Set reasonable math learning goals to motivate active math learning<br>Before engaging in math learning activities, such as group activities and exercises, set goals that you can achieve by making efforts and make continuous efforts to achieve these, thus motivating yourself to learn math actively.<br>3. Actively run for positions such as team leader of mathematics and develop a sense of responsibility in completing math tasks<br>Actively run for positions such as team leader of mathematics. If elected, you should lead your team to complete math tasks on time through effective division

			of labor and cooperation. If not elected, you should actively complete tasks assigned by the team leader as a team member. Develop a sense of responsibility for math learning during the process of carrying out math tasks.
Willpower (45 points)	Self-discipline (25 points)	High-level students (X ≥ 19):<br>1. Highly disciplined. For example, they always finish their homework without being urged by others.<br>2. Not susceptible to distractions.	Well done! Keep it up.
		Intermediate-level students (16 ≤ X < 19):<br>1. Room for improvement still exists in self-discipline.<br>2. Should be less susceptible to distractions.<br>Low-level students (X<16):<br>1. Perform poorly in self-discipline.<br>2. Easily distracted.	1. Make to-do lists to improve self-discipline in math learning<br>Write down and list daily and weekly math learning tasks and goals. Make specific to-do lists and complete each task on time in a structured and planned manner and cross out completed tasks. By getting things done one by one, you can improve self-discipline in math learning.<br>2. Seek out highly disciplined teachers and classmates as role models to motivate yourself to stay disciplined<br>Seek out highly disciplined teachers and classmates as role models, discover and observe their behaviors of self-discipline. For example, teachers who are not late for class and peers who always take the initiative in

			completing their math homework. Learn from them and motivate yourself to stay disciplined.<br>3. Create a good environment for math learning and eliminate the temptation of distractions<br>To create a good environment for math learning and eliminate the temptation of external distractions, such as noise and electronic products. You can take the following two measures. First, try to find a quiet and comfortable environment when learning math. Second, put your cell phone, tablet, game consoles, and so on away.
	Perseverance (20 points)	High-level student ($X \geq 16$):<br>1. Can focus on learning even with external distractions.<br>2. Can effectively handle setbacks in math learning.	Well done! Keep it up.
		Intermediate-level students ($13 \leq X < 16$):<br>1. Perform moderately in focusing on learning when external distractions are present.<br>2. Room for improvement still exists in handling setbacks in math learning.<br>Low-level students ($X<13$):<br>1. Perform poorly in focusing on learning when external distractions are present.	1. Set learning hours and gradually extending these to steadily improve your perseverance in math learning<br>Set math learning hours to ensure completing math learning tasks with efficiency, high quality and quantity within a specified time period. Do not react to external distractions. If you are able to complete the learning hours you have set, gradually extending these to steadily improve your perseverance in math

		2. Display weaknesses in handling setbacks in math learning.	learning.<br>2. Engage in deliberate practice to learn math in a more targeted manner and gain experience in overcoming difficulties<br>Deliberate practice is not simply doing something over and over again; it is target-oriented practice through which you will constantly exceed your current level. During practice, thinking about how to make progress and keep trying is important. With expansive experience accumulated, you will learn to know how to cope with complex situations and have increasingly better performance.<br>3. Exercise with obstacles in a reasonable way to improve your willpower and perseverance<br>Consciously create difficult situations. For instance, you can do some difficult exercises, and participate in challenging mathematical activities, such as mathematical investigations and modeling activities. By dealing with the obstacles in the exercises and explorations, you can exercise with setbacks in math learning. By overcoming difficulties, you can improve your willpower in various aspects.
Personality (50 points)	Query spirit (30 points)	High-level students ($X \geq 23$):<br>1. Skeptical. For example, they dare to question the content of lectures and the concepts in	Well done! Keep it up.

		textbooks.<br>2. Actively ask questions to their teachers or peers to find correct answers when they find problems in math learning.	
		Intermediate-level students ($19 \leq X < 23$):<br>1. Room for improvement exists in terms of skepticism about mathematical knowledge. They are not sufficiently skeptical. For example, they believe that teachers' lectures and textbooks are authoritative. Even if they are confused about something, they do not have the courage to raise a question.<br>2. Room for improvement exists in asking questions about math.<br>Low-level students ($X<19$):<br>1. Perform poorly in terms of skepticism about mathematical knowledge. They lack skepticism. For instance, they believe that teachers and textbooks are authoritative, and they never intend to raise a question.<br>2. They perform poorly in asking questions about math.	1. Learn about the querying spirit of famous mathematicians and understand the importance of such spirit<br>Read more stories about famous mathematicians to understand that many overthrew previous mathematical theories to establish correct theories. For example, Hippasus discovered irrational numbers, overthrowing Pythagoras' theory that only rational numbers exist. By doing so, you will further appreciate the importance of the querying spirit for the development and advancement of mathematics, and for finding correct mathematical ideas.<br>2. Develop a habit of asking questions, cultivate the spirit of getting to the bottom of the matter, and establish confidence in questioning<br>Learn mathematics with questions and actively ask questions in class and when studying after class to develop a habit of asking questions. Do not go too deep into details. Dare to propose different ideas to develop confidence in questioning.<br>3. Reflect on established results in mathematics and

			dare to question the authorities<br>Established results include theorems, laws, equations, textbooks, and results obtained by teachers.<br>Think critically about established results in math, and do not accept these as standards. Ask questions when you are in doubt about established results.
	Competitiveness (20 points)	High-level students (X ≥ 14):<br>1. Actively seek competition and cooperation.<br>2. Work hard to outperform others when falling behind.<br>3. Continuously transcend themselves in math learning.	Well done! Keep it up.
		Intermediate-level students (11 ≤ X < 14):<br>1. Room for improvement exists in seeking competition and cooperation.<br>2. Room for improvement exists in working hard to outperform others.<br>3. Room for improvement exists in active self-transcendence.<br>Low-level students (X<11):<br>1. Perform poorly in seeking competition and cooperation.<br>2. Perform poorly in working hard to outperform others.	1. Actively participate in math activities to experience competition and cooperation<br>According to your own strengths and preferences, actively participate in certain types of mathematical activities. For example, you can raise questions in class and engage in group discussions to demonstrate your abilities by competing and cooperating with others on a fair and reasonable basis. Experience the thrill of competition and the joy of cooperation while participating in math activities.<br>2. Face up to the gaps between themselves and others, take advantage of your strengths and weaknesses, and

		3. Perform poorly in active self-transcendence.	strive to surpass others<br>In mathematics learning, we should face up to the gap between ourselves and others, discover the advantages of others, and admit our own shortcomings. Actively communicate with others about their mathematics learning experience, learn from their strengths, and compensate for your own shortcomings while maintaining your own advantages. Work hard to surpass others in mathematics learning in the future.<br>3. Set reasonable and clear learning goals and strive to surpass yourself<br>Set goals that can be achieved through hard work based on your own level of math knowledge. Use such goals to motivate learning and strive to accomplish that goal. Give yourself approval for every goal you accomplish, increase your self-confidence, be competitive, and strive to surpass yourself.
